# Radiocobalt theranostic applications: current landscape, challenges, and future directions

**DOI:** 10.3389/fnume.2025.1663748

**Published:** 2025-08-06

**Authors:** Alexis M. Sanwick, Ivis F. Chaple

**Affiliations:** Department of Nuclear Engineering, University of Tennessee, Knoxville, TN, United States

**Keywords:** theranostics, positron emission tomography, targeted radionuclide therapy, radiocobalt, cobalt-55, cobalt-58m, auger therapy

## Abstract

Radiocobalt-based theranostics has emerged as a promising platform in nuclear medicine that offers dual capabilities for both diagnostic imaging and targeted radionuclide therapy. ^55^Co (t_1/2_ = 17.53 h, β^+^ = 77%, E*_γ_* = 931.1 keV, I*_γ_* = 75%) and ^58m^Co (t_1/2_ = 9.10 h, IC = 100%) serve as an elementally matched pair for positron emission tomography and targeted Auger electron therapy, respectively, that enable a more personalized approach to cancer management, where imaging with ^55^Co can help to guide and predict therapeutic outcomes for ^58m^Co therapy. The unique coordination chemistry of cobalt allows for stable complexation with various chelators, enhancing *in vivo* stability and targeting efficacy when conjugated to biomolecules such as peptides, antibodies, and small molecules. Recent developments in radiolabeling techniques, chelator design, and preclinical evaluations have significantly improved the pharmacokinetic profiles and tumor specificity of radiocobalt-based radiopharmaceuticals. The aim of this mini review is to provide an overview of the recent advancements and applications of radiocobalt isotopes with a particular focus on the production, chelation chemistry, and *in vivo* targeting of ^55^Co- and ^58m^Co-labelled radiopharmaceuticals over the last 5 years. While challenges still exist in production scalability, dosimetry optimization, and clinical translations, the current trajectory suggests a growing role for radiocobalt-based theranostics in precision oncology.

## 1 Introduction

Theranostics is an emerging medical approach that incorporates paired radiopharmaceuticals to selectively diagnose (“diagnostics”) and treat (“therapeutics”) various types of disease (1, 2). Theranostic radiopharmaceuticals consist of a radionuclide tethered to a vector (antibody, peptide, small molecule, etc.) that binds to a target (3). A positron or gamma-emitting radionuclide can be incorporated for diagnostic imaging with positron emission tomography (PET) or single-photon emitting computed tomography (SPECT) to provide a visual representation of the radiopharmaceutical distribution *in vivo* and help identify specific sites of disease. Diagnostic imaging provides information pertinent to disease staging, identifications of patients who express the specific target, and dosimetry for the radiopharmaceutical. Additionally, diagnostic radiopharmaceuticals can often identify disease before anatomical abnormalities have been determined. Targeted radiopharmaceutical therapy (TRT) incorporates alpha-particle, beta-particle, or Auger electron emitting radionuclides to deliver a therapeutic dose of ionizing radiation to the intended target. Leading treatment modalities including chemotherapy or radiation therapy often result in damage to healthy cells causing debilitating symptoms. TRT leads to radiopharmaceutical accumulation at the target where a cytotoxic radiation dose can be delivered, while minimizing the dose to healthy tissue. The clinical success of theranostics has continued to evolve with the recent U.S. Food and Drug Administration (FDA) approvals of therapeutic ^177^Lu-based radiopharmaceuticals ($t_{1/2}$= 6.7 d, $E_{\beta -}$  = 134 keV) such as [^177^Lu]Lu-DOTATATE (Lutathera) and [^177^Lu]Lu-PSMA-617 (Pluvicto) for treatment of neuroendocrine tumors and prostate cancer, respectively (4–6). These radiopharmaceuticals are often paired with PET radionuclides such as ^68^Ga ($t_{1/2}$= 68 min, $\beta^{+}$=89%, E_max_ = 1,899 keV) or ^64^Cu ($t_{1/2}$= 12.7 h, $\beta^{+}$ = 17.9%, E_max_ = 660 keV) for staging and characterization of disease. One main challenge with radiopharmaceutical development arises in identifying matched pair radionuclides for diagnostic and therapeutic radiopharmaceuticals. Ideally, the chemistry of the diagnostic and therapeutic radionuclides should be identical to ensure that differences in radiopharmaceutical synthesis, binding affinities, off-target binding, biodistribution, etc. do not arise. Several theranostic pairs, including but not limited to, ^61^Cu/^64^Cu/^67^Cu, ^44^Sc/^47^Sc, and ^149^Tb/^152^Tb/^155^Tb/^161^Tb, have garnered significant attention in the field of theranostics as a result of their identical chemistry and complementary nuclear characteristics, enabling both diagnostic imaging and TRT within a single element platform (7–9). Radiocobalt-based theranostics can bridge the gap of the aforementioned shortcomings and provide an additional elementally matched pair for diagnostic imaging (^55^Co) and TRT (^58m^Co). This mini review aims to provide an up-to-date overview of advances and challenges in radiocobalt radiopharmaceutical development in the new decade. Production and purification of radiocobalt for nuclear medicine applications prior to 2021 are omitted from this review as they were described in detail by Barrett et al. (10).

## 2 Properties of medically relevant radiocobalt isotopes

Cobalt is a transition element with atomic number 27 that plays an essential role in oncological medicine with applications including, but not limited to, radiotherapy and diagnostic imaging (11–14). While stable cobalt exists as ^59^Co, 28 radioisotopes of cobalt have been characterized to date, and those radioisotopes that are most applicable for radiopharmaceutical development in nuclear medicine are tabulated in Table 1.

**Table 1:** Decay characteristics, production methods, and applications of radiocobalt isotopes in nuclear medicine.

Isotope	$t_{1/2}$	Decay characteristics	Production	Application	Reference
^55^Co	17.5 h	*β*^+^ = 77%, E_ave__β+_ = 570 keV E*_γ_* = 931.1 keV (75%)	^56^Fe(p,2n)^55^Co ^58^Ni(p,α)^55^Co ^54^Fe(d,n)^55^Co	PET	(15–18, 20, 49)
^ 57^Co	271.8 d	EC = 100%, E_γ_ = 122.06 keV (85.6%)	^58^Ni(p,2p)^57^Co ^58^Fe(p,2n)^57^Co ^57^Fe(d,2n)^57^Co ^60^Ni(p,α)^57^Co ^56^Fe(p,γ)^57^Co	SPECT	(15, 16, 18, 52, 53)
^ 58m^Co	9.10 h	IC = 100%	^58^Fe(p,n)^58m^Co ^57^Fe(d,n)^58m^Co ^61^Ni(p,α)^58m^Co ^55^Mn(α,n)^58m^Co	Auger Therapy	(16, 24, 27)
^ 58g^Co	70.9 d	β^+^ = 14.94%, E_ave__β+_ = 570 keV	^58^Fe(p,n)^58g^Co ^57^Fe(d,n)^58g^Co ^61^Ni(p,α)^58g^Co ^55^Mn(α,n)^58g^Co	PET	(16, 24, 27)

^55^Co is a positron-emitting radiometal of interest for its application as a PET tracer. The high positron branching ratio and half-life compatible for radiolabeling macromolecules can be seen as advantageous over current tracers such as ^18^F ($t_{1/2}$= 110 min), ^64^Cu ($t_{1/2}$= 12.7 h), ^68^Ga ($t_{1/2}$= 68 min), or ^89^Zr ($t_{1/2}$= 78.4 h). ^55^Co is produced primarily through charged particle irradiation with the ^58^Ni(p, α)^55^Co and ^54^Fe(d,n)^55^Co nuclear reactions being the most commonly employed (15–18). Although the ^56^Fe(p,2n)^55^Co reaction has been employed, the production method is limited by the coproduction of ^56^Co and ^57^Co that are chemically inseparable from ^55^Co. The ^54^Fe(d,n)^55^Co reaction has a high cross section at low energies and has the advantage of producing radionuclidically pure ^55^Co by minimizing the coinciding production of ^56^Co and ^57^Co; however, enriched iron targets are necessary for the production of radionuclidic and radioisotopically pure ^55^Co (16, 19, 20). The ^58^Ni(p, α)^55^Co reaction also coproduces ^56^Co and ^57^Co at higher energies (15, 18). Photoproton production via the ^58^Ni(γ,p2n)^55^Co and ^60^Ni(γ,p4n)^55^Co reactions have been attempted, but the radionuclide purity of ^55^Co was deemed insufficient for use in PET as ^56,57,58^Co contaminants were present (21).

^57^Co decays primarily to stable ^57^Fe by electron capture, emitting low energy gamma rays that are suitable for detection via SPECT. The long half-life of ^57^Co (*t*_1/2_ = 271.8 d) can be deemed discouraging for use in diagnostic imaging, although it has been incorporated into some SPECT radiopharmaceuticals (13, 20, 22). The current role of ^57^Co in nuclear medicine is limited to tracer optimization, serving as a surrogate for ^55/58m^Co in the radiopharmaceutical development stage. ^57^Co can be produced by the ^58^Ni(p,2p)^57^Co reaction at energies above 15 MeV, although other production methods are listed in Table 1 (18).

^58m^Co is a promising therapeutic radionuclide with potential applications in Auger electron therapy (23–25). ^58m^Co decays to ^58g^Co via internal conversion and releases 3 low energy Auger electrons, when internalized by a cancerous cell, can directly damage DNA. The decay product, ^58g^Co, is a positron emitter that can potentially be utilized for PET imaging but does emit a prominent 811 keV gamma ray (99.5%) that would deposit unintended dose to normal tissue. ^58m^Co is produced in highest yields by either proton or deuteron bombardment of enriched iron targets, although success using nickel and manganese targets have been demonstrated (24, 26, 27). Since deuterons have a higher stopping power, the targets require a smaller amount of isotopically enriched target material, although significant costs are noted with the target materials. While other radioisotopic impurities can be reduced with modification to charged particle energies and target enrichment, the co-production of ^58g^Co is unavoidable. Valdovinos et al. evaluated the activity yields for the production of ^58m^Co and ^58g^Co via the ^57^Fe(d,n)^58g^Co reaction. Using deuteron currents between 90 and 124 μ Ah, they reported ^58m^Co yields ranging from 11 to 17.8 MBq/μ Ah and ^58g^Co co-contaminant yields between 115 and 158 MBq/μ Ah at the end of bombardment (16).

## 3 Chelation chemistry

Recent advances in chelation chemistry for radiocobalt, while limited, have concentrated on optimizing coordination stability and redox compatibility to support its use in theranostic applications. The intermediate ionic radius and accessible oxidation states of cobalt including Co^2+^ and Co^3+^ have directed research toward macrocyclic chelators including DOTA and NOTA, along with tailored derivatives like NO2A and NO3A (28). Recent work by Lin et al. has emphasized the importance of controlling cobalt redox chemistry to minimize *in vivo* transchelation and off-target distribution. This study demonstrated that the oxidation state of ^nat/55^Co can be readily modulated and is highly sensitive to the chelator environment. Using the chelator-tryptophan conjugates [^55^Co][Co(NO2A)]-W, [^55^Co][Co(NOTA)]-W and [^55^Co][Co(DO3A)]-W, the authors discovered that the conjugates all readily formed stable Co^2+^ species. However, the NOTA and NO2A conjugates could be readily oxidized to the Co^3+^ state upon exposure to hydrogen peroxide, illustrating the ligand-specific redox behavior pertinent to the radiocobalt complex design (28). Another study by Lin et al. investigated the labeling conditions of ^55^Co with sarcophagine-based chelators. The authors demonstrated that the [^55^Co]Co-DiAmSar complexes could be efficiently formed under mild conditions with purity and stability suitable for pre-clinical *in vivo* radiopharmaceutical applications (29). These results highlight the potential of sarcophagine frameworks for radiocobalt theranostic applications, offering an alternative to traditional macrocyclic chelators with favorable stability profiles. Ge et al. explored the use of a [(triazol-4-yl)methyl]-N1,N2,N2-tris(pyridin-2-ylmethyl)ethane-1,2-diamine (TZTPEN) conjugate for binding Co^2+^ and Co^3+^. While the TZTPEN complex demonstrated promising coordination properties, *in vivo* evaluation revealed bone uptake, indicating potential dissociation and incomplete kinetic stability under physiological conditions. This effort was the first application of a polydentate polypyridyl-amine chelator in radiochemistry (30). The results of these studies will be further discussed in the next section.

## 4 Radiopharmaceutical development

The discovery of molecular targets and the development of suitable chelators for targeted imaging and therapy remain active areas of preclinical research. This section offers a concise overview of molecular targets explored using ^55^Co- and ^58m^Co-labeled radiopharmaceuticals for theranostic applications since 2021.

### 4.1 Neurotensin receptors

Neurotensin receptors (NTSRs) are a family of transmembrane G-protein-coupled receptors (GPCR) that bind the peptide neurotensin (NTS), which acts as a neurotransmitter or neuromodulator in the central nervous system and as a hormone in the peripheral nervous system. NTS promotes the growth of tissue, suggesting that NTS could contribute to the growth of cancers (31–33). Three subtypes of NTSRs have been identified and are overexpressed in varying levels in different types of cancers including pancreatic, prostate, breast, colon, and non-small cell lung cancers, indicating that it may be a suitable molecular target (34–38). A theranostic study using novel [^55/58m^Co]Co-NOTA-NT-20.3 tracers investigated the targeting properties and cytotoxicity in HT29 human colorectal adenocarcinoma cells. *in vitro* experiments using [^55/58m^Co]Co-NOTA-NT-20.3 suggested that the tracer behaves similarly to natural neurotensin with reduced binding affinity. *in vivo* imaging with [^55^Co]Co-NOTA-NT-20.3 showed uptake in the HT29 tumor, indicating radiotracer selectivity (25, 39). While therapy studies demonstrated enhanced cytotoxicity of [^58m^Co]Co-NOTA-NT-20.3 in comparison to [^58m^Co]CoCl_2_ (>15x), only modest therapeutic response was identified *in vivo*, and the tumor receiving an absorbed dose of 0.6 Gy from a 110 MBq treatment. Due to the low absorbed dose to the tumor, the authors suggested that uptake could be enhanced by utilizing alternative neurotensin analogues with improved binding affinities or by employing neurotensin antagonists (25). However, it is crucial that any selected alternative retains internalizing capability, particularly to the nucleus, as it is essential for Auger therapy.

Another study investigated the *in vivo* feasibility of a ^55^Co-labeled NTS complex conjugated with a functionalized sarcophagine chelator and found that [^55^Co]Co-NT-sarcage demonstrated prominent tumor uptake in HT29 xenografted nude mice and achieved a high tumor-to-background ratio (29). Fonseca Cabrera et al. synthesized ^64^Cu-, ^55^Co-, and ^68^Ga-labeled radiopharmaceuticals targeting different tumor models expressing NTSR-1 and investigated the effect of macrocycles on *in vivo* distribution. [^55^Co]Co-NT-CB-NOTA revealed high tumor uptake, high tumor-to-background contrast, and sustained tumor uptake in HT29 tumor models leading the authors to conclude that NT-Sarcage labeled with ^55^Co/^58m^Co may be an excellent theranostic pair targeting NTSR-1 positive cancers (40).

### 4.2 Prostate-Specific membrane antigen

**Figure 1:**
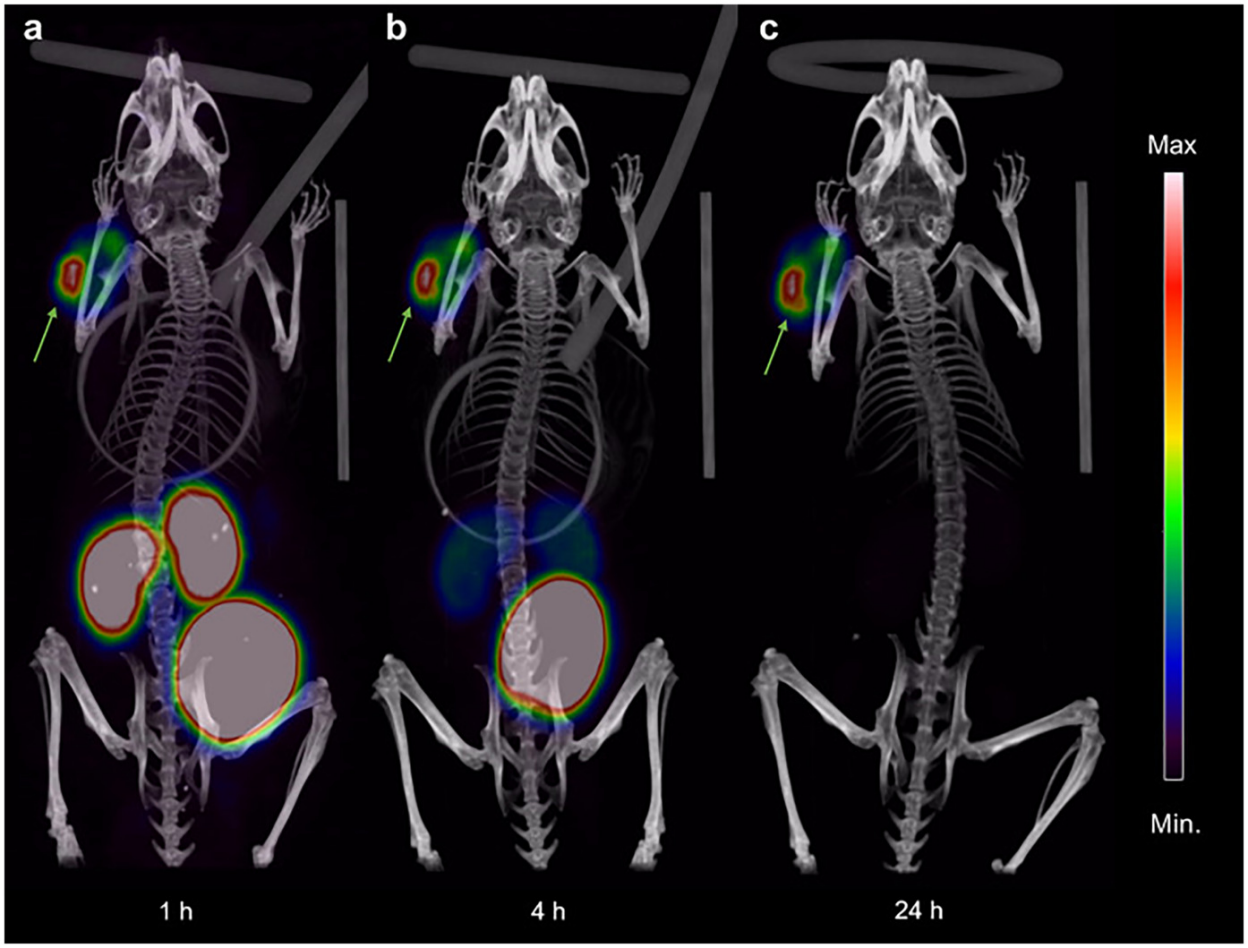
Coronal maximum intensity projection PET/CT images showing uptake and distribution of [^55^Co]Co-DOTA-PSMA-617 in PC3-PIP tumor-bearing NOD-SCID mice at **(a)** 1 h, **(b)** 4 h, and **(c)** 24 h pi. The intensity of the PET data is displayed from zero to maximum tumor uptake. Arrows indicate the subcutaneous PC3-PIP tumor. [Figure adapted from Baun et al. (23) Creative Commons Attribution 4.0 International License].

Prostate-specific membrane antigen (PSMA) is a type II membrane glycoprotein that is overexpressed in prostate cancer and has been deemed the most promising target for prostate cancer imaging and therapy, to date, as highlighted via the FDA approval of [^177^Lu]Lu-PSMA-617 (41). A preclinical study by Baun et al. evaluated the theranostic pair [^55/58m^Co]Co-DOTA-PSMA-617 for PET imaging and Auger therapy of prostate cancer using PC3-PIP (PSMA+), LNCaP (PSMA+), and PC3-flu (PSMA-) cell lines. *in vitro* binding assays showed PSMA-specific uptake in PC3-PIP and LNCaP cells with high cell-associated activity in the nucleus. *in vivo* studies in tumor-bearing mice showed high specific tumor uptake and a significantly increased median survival for mice treated with [^58m^Co]Co-DOTA-PSMA-617 compared to control mice (Figure 1). The authors concluded that [^55/58m^Co]Co-DOTA-PSMA-617 exhibited excellent *in vitro* and *in vivo* properties with no observed toxicities (23). A deep dive into the synthesis of single redox species employed the PSMA-targeted radiopharmaceuticals [^55^Co]Co-NO2A-PSMA-617, [^55^Co]Co-NOTA-PSMA-617, and [^55^Co]Co-DO3A-PSMA-617 to stabilize ^55^Co^2+^ or ^55^Co^3+^ species for PET imaging in a mouse model. Results of the PET region of interest quantification and the biodistribution profiles at 24 h post-injection of the three tracers identified [^55^Co]Co-DO3A-PSMA-617 as achieving the highest tumor uptake and suggested that it may be due to the anionic charge of the complex. They determined that once the ^55^Co^2+^ and ^55^Co^3+^ species are formed and isolated as thermodynamically favored, the oxidation state is retained *in vivo* and suitable for radiopharmaceutical development (28).

### 4.3 Gastrin-releasing peptide receptors

Gastrin-releasing peptide receptors (GRPRs) are compelling molecular targets for imaging and therapy due to their overexpression in a variety of malignancies, including prostate, gastrointestinal, and breast cancers (42–44). Targeting GRPRs with PET imaging agents has demonstrated the potential to enhance imaging sensitivity and facilitate the early detection of lymph node involvement. Notably, GRPR has emerged as a promising target for estrogen-receptor positive (ER+) breast cancer in a preclinical study using [^55^Co]Co- and [^177^Lu]Lu-DOTA-RM26. *in vitro* studies using the ER + T47D cell line demonstrated high binding that significantly increased from 1 h to 4 h and was effectively blocked by a 1,000× excess of unlabeled DOTA-RM26, confirming binding specificity. *in vivo* biodistribution studies revealed that the tumor exhibited the highest radioligand uptake at 4 h post-injection, followed by the kidneys. Although tumor uptake decreased over time, it remained the highest uptake among all tissues. The authors concluded PET/CT imaging with [^55^Co]Co-DOTA-RM26 demonstrated GRPR-specific tumor visualization and enabled next day imaging (44).

### 4.4 Human epidermal growth factor receptors

The human epidermal growth factor receptors (HER) are a family of transmembrane proteins involved in regulating proliferation, motility, and apoptosis (45). Overexpression of the epidermal growth factor receptor (EGFR/HER1) is implicated in several cancers, including non-small-cell lung cancer and head and neck squamous cell carcinoma, making EGFR a well-established target for anti-cancer therapies (46–48). [^57^Co]Co-GE11-TZTPEN is a novel construct that was investigated for its EGFR-targeting capabilities by Ge et al. *in vitro* studies confirmed a high radiochemical yield (99%) and radionuclidic purity (99%), along with specific uptake in EGFR-positive cell lines. However, *in vivo* imaging revealed negligible tumor uptake and predominant accumulation in non-target organs, including the liver, gall bladder, kidneys, and spleen. Based on these findings, [^57^Co]Co-GE11-TZTPEN was deemed unsuitable as an EGFR-targeted radiopharmaceutical for *in vivo* applications (30).

## 5 Challenges and future directions

While radiocobalt-based theranostics offer unique advantages for PET imaging and targeted Auger electron therapy, particularly due to the favorable half-lives of ^55^Co ($t_{1/2}$ = 17.5 h) and ^58m^Co ($t_{1/2}$ = 9.1 h), and their strong coordination chemistry, several key challenges remain that hinder clinical translation. One of the major limitations is the coproduction of long-lived radionuclidic contaminants during cyclotron irradiation including ^56^Co ($t_{1/2}$ = 77.2 d) and ^58g^Co ($t_{1/2}$ = 70.9 d). These impurities are particularly problematic for therapeutic applications, where small contributions can substantially increase patient dose and compromise safety.

A significant barrier to radiocobalt production is the need for medium or high energy cyclotrons and enriched target materials, such as ^58^Ni and ^54^Fe, which are expensive and not widely available. One solution involves implementation of target recovery and recycling methods as demonstrated by Valdovinos et al., which could reduce the cost of production (16). Additionally, alternative target designs could improve manufacturability. Siikanen et al. designed a target consisting of a ^58^Ni/Mg matrix that enabled faster target fabrication, higher thermal conductivity, and a more efficient dissolution to streamline the production of ^55^Co (49). Another study investigated the employment of a siphon style liquid target system to produce ^58m^Co on a 13 MeV medical cyclotron (50). Improved target design and reprocessing could support routine clinical production leading to more widespread use.

Despite growing interest in the field of theranostics, there are currently no FDA-approved radiopharmaceuticals incorporating radiocobalt. While preclinical studies have demonstrated that ^55/58m^Co-labeled analogues can offer superior imaging and therapeutic capabilities compared to some FDA-approved agents incorporating ^68^Ga and ^64^Cu, further research is needed to identify predictive biomarkers that support clinical efficacy (51). To highlight an example of such, radiocobalt-labeled NTS analogues have demonstrated excellent *in vitro* cytotoxicity, but only modest therapeutic effects were observed *in vivo*, highlighting the need for biomarkers that better correlate with therapeutic outcomes (25). In contrast, PSMA-targeted imaging and therapy with ^55/58m^Co-labeled analogues have demonstrated excellent internalization and tumor uptake, indicating the potential for clinical translation (23). Additionally, ^55^Co-labeled compounds targeting GRPRs and EGFRs have allowed for successful visualization of tumors in mouse models, though therapeutic data utilizing ^58m^Co remains limited and should be the priority for future work.

To advance clinical potential, improvements in *in vivo* performance should focus on improving the stability and minimizing off-target uptake. This highlights the need for novel chelators that exhibit fast complexation kinetics and maintain high *in vivo* stability. Expanding the range of molecular targets and designing radiopharmaceuticals with high binding affinities is critical for broadening the scope of radiocobalt-based theranostics. However, clinical translation may face considerable challenges when considering the need for dosimetric validations and toxicity profiling.

## 6 Conclusion

The radiocobalt isotopes ^55^Co and ^58m^Co offer a promising elementally matched pair for theranostic applications due to their excellent nuclear properties and identical coordination chemistry. This theranostic pair supports the development of novel radiopharmaceuticals for PET imaging (^55^Co) and Auger electron therapy (^58m^Co), with the capability targeting a wide variety of malignancies. To date approximately 67 papers have been published on ^55^Co and 12 on ^58m^Co, with 12 and 8 of those, respectively, having been published in the last five years. This indicates a trending increase in the excitement towards the production, radiochemistry, and translation of radiocobalt. Successful clinical translation relies on robust isotope production methods and the formulation of stable radiopharmaceuticals with a high affinity and specificity for molecular targets of interest. Although numerous production methods have been identified, proton and deuteron irradiation of enriched targets remain the most commonly implemented and have demonstrated the ability to generate high purity ^55^Co and ^58m^Co suitable for radiopharmaceutical use. These production methods are mostly limited by the limited supply and expense of enriched target materials and access to cyclotrons. Future efforts should prioritize on enhancing production efficiency while maintaining high radionuclidic purity and cost-effectiveness. Advancements in chelation chemistry and the development of novel targeting vectors have led to promising preclinical results in prostate, breast, and other cancers, however, challenges still persist in achieving high *in vivo* stability, reducing off-target accumulation, and validating therapeutic efficacy in preclinical studies. Radiocobalt theranostics provides a compelling platform in precision medicine. Continued progress in isotope production and radiopharmaceutical design are essential in bridging the gap between preclinical studies and clinical translation.

## References

[1] Jeelani S Reddy RC Maheswaran T Asokan GS Dany A Anand B. Theranostics: a treasured tailor for tomorrow. J Pharm Bioallied Sci. (2014) 6(Suppl 1):S6–8. 10.4103/0975-7406.137249 25210387 PMC4157283

[2] Burkett BJ Bartlett DJ McGarrah PW Lewis AR Johnson DR Berberoglu K A review of theranostics: perspectives on emerging approaches and clinical advancements. Radiol Imaging Cancer. (2023) 5(4):e220157. 10.1148/rycan.220157 37477566 PMC10413300

[3] Vahidfar N Aghanejad A Ahmadzadehfar H Farzanehfar S Eppard E. Theranostic advances in breast cancer in nuclear medicine. Int J Mol Sci. (2021) 22(9):4597. 10.3390/ijms22094597 33925632 PMC8125561

[4] Hennrich U Eder M. [^177^lu]Lu-PSMA-617 (Pluvicto™): the first FDA-approved radiotherapeutical for treatment of prostate cancer. Pharmaceuticals. (2022) 15(10):1292. 10.3390/ph15101292 36297404 PMC9608311

[5] Hennrich U Kopka K. Lutathera((R)): the first FDA- and EMA-approved radiopharmaceutical for peptide receptor radionuclide therapy. Pharmaceuticals (Basel). (2019) 12(3):114. 10.3390/ph12030114 31362406 PMC6789871

[6] Rohith G. VISION Trial: (177)Lu-PSMA-617 for progressive metastatic castration-resistant prostate cancer. Indian J Urol. (2021) 37(4):372–3. 10.4103/iju.iju_292_21 34759536 PMC8555571

[7] Bailey DL Willowson KP Harris M Biggin C Aslani A Lengkeek NA ^64^Cu Treatment planning and ^67^Cu therapy with radiolabeled [^64^Cu/^67^Cu]MeCOSar-octreotate in subjects with unresectable multifocal meningioma: initial results for human imaging, safety, biodistribution, and radiation dosimetry. J Nucl Med. (2023) 64(5):704. 10.2967/jnumed.122.264586 36460344

[8] Van Laere C Koole M Deroose CM de Voorde MV Baete K Cocolios TE Terbium radionuclides for theranostic applications in nuclear medicine: from atom to bedside. Theranostics. (2024) 14(4):1720–43. 10.7150/thno.92775 38389843 PMC10879862

[9] Müller C Domnanich KA Umbricht CA van der Meulen NP. Scandium and terbium radionuclides for radiotheranostics: current state of development towards clinical application. Br J Radiol. (2018) 91(1091):20180074. 10.1259/bjr.20180074 29658792 PMC6475947

[10] Barrett KE Houson HA Lin W Lapi SE Engle JW. Production, purification, and applications of a potential theranostic pair: cobalt-55 and cobalt-58m. Diagnostics (Basel). (2021) 11(7):1235. 10.3390/diagnostics11071235 34359318 PMC8306844

[11] Jansen HM Paans AM vd Vliet AM Veenma-van der Duin L Bolwijn-Meijer CJ Pruim J Cobalt-55 positron emission tomography in ischemic stroke. Clin Neurol Neurosurg. (1997) 99(1):6–10. 10.1016/S0303-8467(96)00558-6 9107460

[12] Front D Israel O Even-Sapir E Iosilevsky G Frenkel A Ben-Haim S The concentration of bleomycin labeled with co-57 in primary and metastatic tumors. Cancer. (1989) 64(5):988–93. 10.1002/1097-0142(19890901)64:5<988::AID-CNCR2820640503>3.0.CO;2-S 2474369

[13] Front D Israel O Iosilevsky G Even-Sapir E Frenkel A Peleg H Human lung tumors: sPECT quantitation of differences in co-57 bleomycin uptake. Radiology. (1987) 165(1):129–33. 10.1148/radiology.165.1.2442794 2442794

[14] Schreiner LJ Joshi CP Darko J Kerr A Salomons G Dhanesar S. The role of cobalt-60 in modern radiation therapy: dose delivery and image guidance. J Med Phys. (2009) 34(3):133–6. 10.4103/0971-6203.54846 20098559 PMC2807677

[15] Mastren T Marquez BV Sultan DE Bollinger E Eisenbeis P Voller T Cyclotron production of high-specific activity 55Co and *in vivo* evaluation of the stability of 55Co metal-chelate-peptide complexes. Mol Imaging. (2015) 14(10):526–33. 10.2310/7290.2015.00025 26505224 PMC4863226

[16] Valdovinos HF Hernandez R Graves S Ellison PA Barnhart TE Theuer CP Cyclotron production and radiochemical separation of (55)Co and (58 m)Co from (54)Fe, (58)Ni and (57)Fe targets. Appl Radiat Isot. (2017) 130:90–101. 10.1016/j.apradiso.2017.09.005 28946101 PMC5673506

[17] Valdovinos HF Graves S Barnhart T Nickles RJ. 55Co Separation from proton irradiated metallic nickel. AIP Conf Proc. (2014) 1626(1):217–20. 10.1063/1.4901397

[18] Spellerberg S Reimer P Blessing G Coenen HH Qaim SM. Production of 55Co and 57Co via proton induced reactions on highly enriched 58Ni. Appl Radiat Isot. (1998) 49(12):1519–22. 10.1016/S0969-8043(97)10119-1

[19] Zaman MR Qaim SM. Excitation functions of (d,n) and (d,*α*) reactions on 54Fe: relevance to the production of high purity 55Co at a small cyclotron. Radiochim Acta. (1996) 75(2):59–64. 10.1524/ract.1996.75.2.59

[20] Sharma H Zweit J Smith AM Downey S. Production of cobalt-55, a short-lived, positron emitting radiolabel for bleomycin. Int J Rad Appl Instrum A. (1986) 37(2):105–9. 10.1016/0883-2889(86)90055-9 2428771

[21] Kazakov AG Babenya JS Ekatova TY Belyshev SS Khankin VV Kuznetsov AA Yields of photo-proton reactions on nuclei of nickel and separation of cobalt isotopes from irradiated targets. Molecules. (2022) 27(5):1524. 10.3390/molecules27051524 35268626 PMC8911929

[22] Doscherholmen A. Plasma absorption of cyanocobalamin co 57: diagnostic value in vitamin B12 malabsorption states. Arch Intern Med. (1974) 134(6):1019–24. 10.1001/archinte.1974.00320240053005 4473962

[23] Baun C Dam JH Hildebrandt MG Ewald JD Kristensen BW Gammelsrod VS Preclinical evaluation of [(58 m)Co]co-DOTA-PSMA-617 for auger electron therapy of prostate cancer. Sci Rep. (2023) 13(1):18837. 10.1038/s41598-023-43429-8 37914790 PMC10620164

[24] Thisgaard H Elema DR Jensen M. Production and dosimetric aspects of the potent auger emitter 58mCo for targeted radionuclide therapy of small tumors. Med Phys. (2011) 38(8):4535–41. 10.1118/1.3608905 21928624

[25] Lin W Aluicio-Sarduy E Houson HA Barnhart TE Tekin V Jeffery JJ Theranostic cobalt-55/58 m for neurotensin receptor-mediated radiotherapy *in vivo*: a pilot study with dosimetry. Nucl Med Biol. (2023) 118-119:108329. 10.1016/j.nucmedbio.2023.108329 36805869 PMC10121947

[26] Thisgaard H Olsen BB Dam JH Bollen P Mollenhauer J Hoilund-Carlsen PF. Evaluation of cobalt-labeled octreotide analogs for molecular imaging and auger electron-based radionuclide therapy. J Nucl Med. (2014) 55(8):1311–6. 10.2967/jnumed.114.137182 24876207

[27] Sudár S Qaim SM. Isomeric cross-section ratio for the formation of 58Com,g in neutron, proton, deuteron, and alpha-particle induced reactions in the energy region up to 25 MeV. Phys Rev, C Nucl Phys. (1996) 53(6):2885–92. 10.1103/PhysRevC.53.2885 9971275

[28] Lin W Smilowicz D Joaqui-Joaqui MA Bera A Zhong Z Aluicio-Sarduy E Controlling the redox chemistry of cobalt radiopharmaceuticals. Angew Chem Int Ed Engl. (2024) 63(50):e202412357. 10.1002/anie.202412357 39312186 PMC11609885

[29] Lin W Fonseca Cabrera GO Aluicio-Sarduy E Barnhart TE Mixdorf JC Li Z Radiolabeling diaminosarcophagine with cyclotron-produced cobalt-55 and [(55)Co]co-NT-sarcage as a proof of concept in a murine Xenograft model. Bioconjug Chem. (2024) 35(3):412–8. 10.1021/acs.bioconjchem.4c00043 38411531 PMC10954389

[30] Gé LG Danielsen MB Nielsen AY Skavenborg ML Langkjær N Thisgaard H Radiocobalt-Labeling of a polypyridylamine chelate conjugated to GE11 for EGFR-targeted theranostics. Molecules. (2025) 30(2):212. 10.3390/molecules30020212 39860082 PMC11767697

[31] Carraway R Leeman SE. The isolation of a new hypotensive peptide, neurotensin, from bovine hypothalami. J Biol Chem. (1973) 248(19):6854–61. 10.1016/S0021-9258(19)43429-7 4745447

[32] Ouyang Q Zhou J Yang W Cui H Xu M Yi L. Oncogenic role of neurotensin and neurotensin receptors in various cancers. Clin Exp Pharmacol Physiol. (2017) 44(8):841–6. 10.1111/1440-1681.12787 28556374

[33] Wu Z Martinez-Fong D Tredaniel J Forgez P. Neurotensin and its high affinity receptor 1 as a potential pharmacological target in cancer therapy. Front Endocrinol (Lausanne). (2013) 3:184. 10.3389/fendo.2012.00184 23335914 PMC3547287

[34] Ocejo-Garcia M Ahmed SI Coulson JM Woll PJ. Use of RT-PCR to detect co-expression of neuropeptides and their receptors in lung cancer. Lung Cancer. (2001) 33(1):1–9. 10.1016/S0169-5002(00)00248-8 11429190

[35] Swift SL Burns JE Maitland NJ. Altered expression of neurotensin receptors is associated with the differentiation state of prostate cancer. Cancer Res. (2010) 70(1):347–56. 10.1158/0008-5472.CAN-09-1252 20048080

[36] Maoret JJ Pospai D Rouyer-Fessard C Couvineau A Laboisse C Voisin T Neurotensin receptor and its mRNA are expressed in many human colon cancer cell lines but not in normal colonic epithelium: binding studies and RT-PCR experiments. Biochem Biophys Res Commun. (1994) 203(1):465–71. 10.1006/bbrc.1994.2205 7521165

[37] Wang L Friess H Zhu Z Graber H Zimmermann A Korc M Neurotensin receptor-1 mRNA analysis in normal pancreas and pancreatic disease. Clin Cancer Res. (2000) 6(2):566–71. 10690540

[38] Dupouy S Doan VK Wu Z Mourra N Liu J De Wever O Activation of EGFR, HER2 and HER3 by neurotensin/neurotensin receptor 1 renders breast tumors aggressive yet highly responsive to lapatinib and metformin in mice. Oncotarget. (2014) 5(18):8235–51. 10.18632/oncotarget.1632 25249538 PMC4226680

[39] Houson HA Tekin V Lin W Aluicio-Sarduy E Engle JW Lapi SE. PET Imaging of the neurotensin targeting peptide NOTA-NT-20.3 using cobalt-55, copper-64 and gallium-68. Pharmaceutics. (2022) 14(12):2724. 10.3390/pharmaceutics14122724 36559218 PMC9781609

[40] Fonseca Cabrera GO Ma X Lin W Zhang T Zhao W Pan L Synthesis of ^64^Cu-, ^55^Co-, and ^68^Ga-labeled radiopharmaceuticals targeting neurotensin receptor-1 for theranostics: adjusting *in vivo* distribution using multiamine macrocycles. J Nucl Med. (2024) 65(8):1250. 10.2967/jnumed.124.267469 38871388 PMC11294072

[41] Eiber M Fendler WP Rowe SP Calais J Hofman MS Maurer T Prostate-Specific membrane antigen ligands for imaging and therapy. J Nucl Med. (2017) 58(Suppl 2):67S–76. 10.2967/jnumed.116.186767 28864615

[42] Markwalder R Reubi JC. Gastrin-releasing peptide receptors in the human prostate: relation to neoplastic transformation. Cancer Res. (1999) 59(5):1152–9. 10070977

[43] Reubi JC Körner M Waser B Mazzucchelli L Guillou L. High expression of peptide receptors as a novel target in gastrointestinal stromal tumours. Eur J Nucl Med Mol Imaging. (2004) 31(6):803–10. 10.1007/s00259-004-1476-2 14985869

[44] Baun C Olsen BB Alves CML Ditzel HJ Terp M Hildebrandt MG Gastrin-releasing peptide receptor as theranostic target in estrogen-receptor positive breast cancer: a preclinical study of the theranostic pair [^55^Co]Co- and [^177^Lu]Lu-DOTA-RM26. Nucl Med Biol. (2024) 138-139:108961. 10.1016/j.nucmedbio.2024.108961 39357076

[45] Avraham R Yarden Y. Feedback regulation of EGFR signalling: decision making by early and delayed loops. Nat Rev Mol Cell Biol. (2011) 12(2):104–17. 10.1038/nrm3048 21252999

[46] Ang KK Berkey BA Tu X Zhang HZ Katz R Hammond EH Impact of epidermal growth factor receptor expression on survival and pattern of relapse in patients with advanced head and neck carcinoma. Cancer Res. (2002) 62(24):7350–6. 12499279

[47] Nair S Bonner JA Bredel M. EGFR Mutations in head and neck squamous cell carcinoma. Int J Mol Sci. (2022) 23(7):3818. 10.3390/ijms23073818 35409179 PMC8999014

[48] Prabhakar CN. Epidermal growth factor receptor in non-small cell lung cancer. Transl Lung Cancer Res. (2015) 4(2):110–8. 10.3978/j.issn.2218-6751.2015.01.01 25870793 PMC4384217

[49] Siikanen J Milton S Bratteby K Lin W Engle JW Jussing E Rapid fabrication and dissolution of pressed 58Ni/mg matrix targets for 55Co production. EJNMMI Radiopharma Chem. (2025) 10(1):4. 10.1186/s41181-024-00324-5 PMC11751214 39836306

[50] Mues genannt Koers L McNeil SW Radchenko V Paulssen E Hoehr C. Production of co-58 m in a siphon-style liquid target on a medical cyclotron. Appl Radiat Isot. (2023) 195:110734. 10.1016/j.apradiso.2023.110734 36863263

[51] Andersen TL Baun C Olsen BB Dam JH Thisgaard H. Improving contrast and detectability: imaging with [(55)Co]Co-DOTATATE in Comparison with [(64)Cu]Cu-DOTATATE and [(68)Ga]Ga-DOTATATE. J Nucl Med. (2020) 61(2):228–33. 10.2967/jnumed.119.233015 31519803 PMC8801948

[52] Lee JY Chae J Lee J Hwang I Hur MG Park JH. Production of cobalt-57 for industrial and medical applications in RFT-30 cyclotron facility. J Radioanal Nucl Chem. (2023) 332(12):5097–103. 10.1007/s10967-023-08978-2

[53] O'Brien BJ Coote GE. A study of 57Co by the 56Fe(p, *γ*)57Co reaction. Nucl Phys A. (1970) 153(2):593–609. 10.1016/0375-9474(70)90794-3

